# An Analytical Model for Cure-Induced Deformation of Composite Laminates

**DOI:** 10.3390/polym14142903

**Published:** 2022-07-17

**Authors:** Xiaobo Peng, Jiang Xu, Yong Cheng, Long Zhang, Jie Yang, Yinghui Li

**Affiliations:** 1 School of Mechanics and Aerospace Engineering, Southwest Jiaotong University, Chengdu 610031, China; yuongpeng@sina.com (X.P.); jxu@nwpu.com (J.X.); yhli2007@sina.com (Y.L.); 2 AVIC Chengdu Aircraft Industry (Group) Co., Ltd., Chengdu 610091, China; yong_cheng2011@163.com (Y.C.); zhanglong19523@sina.com (L.Z.); 3 Applied Mechanics and Structure Safety Key Laboratory of Sichuan Province, Southwest Jiaotong University, Chengdu 610031, China

**Keywords:** composite laminates, polymer matrix composites, cure behavior, analytical modelling

## Abstract

Curing deformation prediction plays an important role in guiding the tools, curing process design, etc. Analytical methods can provide a rapid prediction and in-depth understanding of the curing deformation mechanism. In this paper, an analytical model is presented to study the cure-induced deformation of composite laminates. Based on the classical laminate theory, the thermal stress and deformation of composites during the curing process are calculated by considering the evolution of the mechanical properties of resin. Additionally, the coupling stiffness of the laminate is taken into consideration in the analytical model. An interface layer between the tool and the part is developed to simulate the variation of the tool–part interaction with the degree of resin cure. The maximum curing deformations and deformation profiles of different lay-up composite parts predicted by the proposed model are compared with the results of the finite element method and previous literature reports. Then, a comprehensive parametric study is carried out to investigate the influence of curing cycle, geometry, tool thermal expansion, and resin characteristics on the curing deformation of composite parts. The results reveal that geometry has a significant influence on the curing deformation of composite parts, but for dimensionally determined parts, curing deformation is mainly attributable to their own anisotropy in macro and micro aspects, as well as the stretching effect of the tool on the part. The percentage contribution of different factors to curing deformation composites with different lay-ups and geometries is also discussed.

## 1. Introduction

Fiber-reinforced resin matrix composite laminates are commonly used in the aerospace and transportation sectors because of their properties of high specific modulus, high specific strength, excellent fatigue resistance, and excellent designability [1,2,3]. Their application is limited by the curing deformation induced by tool–part interaction, chemical shrinkage of resin, thermal expansion mismatch between the fiber and the resin, and processing defects [4]. The ability to predict curing deformation is essential for the precise manufacture of composite structures in dimensional terms.

In recent decades, numerous experiments have been carried out to observe warpage in composites and study the relationship between curing deformation and the curing process, tools, etc. [5,6,7,8]. Considering the importance of experimental research, Liu et al. [9] carried out several experimental studies to investigate the effect of heating rates and first dwell time on the curing deformation of thermoset composite laminates. Yu et al. [10] designed a series of experiments to investigate residual stresses in epoxy-steel systems due to curing shrinkage, varying thermal shrinkage and hygroscopic expansion, and obtained the relationship between residual stresses and deformation. An experimental study was presented by Fernlund et al. [11], which showed that the geometry of polymer composites after curing is heavily influenced by the curing cycle, tool surface, part geometry, and lay-up. Within the past two years, Chen [12,13] conducted several experimental studies on the effect of cure path on the elastic properties and interlaminar fracture toughness of interlayer-toughened thermoset composites. Oliveira et al. [14] investigated influences of four types of tools, including aluminum, steel, carbon composites and carbon foam, on the internal stresses of carbon fiber composites during autoclave curing using embedded fiber Bragg gratings. Moretti et al. [15] used an experimental approach to examine the effect of curing processes, such as co-curing, co-bonding, and secondary bonding, on the geometric stability of composite laminates. However, composite curing experiments are often costly, time-consuming, and not conducive to parametric studies.

The finite element method (FEM) is widely employed in the prediction of curing deformation, particularly for complicated composite structures [16,17]. Parambil et al. [18] developed a computational FEM incorporating a resin constitutive model for carbon fiber/thermoplastic composites at the microscale. Mezeix et al. [19] used explicit nonlinear FEM to predict the spring-back of composite aircraft structures, with consideration of the physical mechanism of spring-back, such as inter-ply sliding and tool–part interface characteristics. Kim et al. [20] adopted a finite element-based cure simulation to forecast the curing behavior and material properties of thermoset fabrics and investigated the effect of fabric parameters on the deformation induced by the curing process of plain woven composite structures. Qiao et al. [21] studied the curing deformation of complex-shape composite parts using a 3D numerical model considering the influence of tool–part interaction. Kawagoe et al. [22] predicted process-induced deformation of laminates due to curing and thermal shrinkage using a multi-scale model consisting of microscopic and macroscopic finite element analysis, taking into account material and geometric nonlinearities. Luo et al. [23] developed an integrated methodology based on thermodynamic FEM and an artificial neural network to rapidly predict the process-induced shapes of composite laminates. In order to accurately predict curing deformations for finite element analysis, several models of tool–part interaction [24] and constitutive models of resin [25,26,27,28,29,30,31,32,33,34] have also been proposed. However, the finite element method has a few shortcomings in understanding the mechanism of curing deformation of composite materials and explaining the resulting phenomena.

Compared with experimental and finite element methods, closed-form analytical models provide convenient, rapid prediction, and in-depth understanding of the mechanisms of curing deformation within acceptable accuracy. However, a few studies have used analytical models to investigate the curing deformation of composite laminates. Yuan et al. [35], Sun et al. [36], and Arafath et al. [37] proposed an analytical model to predict the curing deformation of a composite part, taking into consideration the tool–part interaction and evolution of material. Twigg et al. [38] proposed a simple analytical model that took account of tool–part interaction as a guide for predicting warpage of laminates. Based on classical laminate theory (CLT), Abouhamzeh et al. [39] presented an analytical model to predict the warpage and residual stresses of fiber metal laminates. Wisnom et al. [40] developed a new analytical solution for the spring-in of curved thermoset matrix composites, which provides new insight into the physical mechanism that causes curved composites to spring in. The analytical solution takes into account the low shear stiffness of the material in the rubbery state before it is fully cured. Che et al. [41] presented an analytical model that considers slip effects from an energy perspective and describes the mechanism of slippage interaction on the curing deformation of fiber metal laminates. However, the coupling stiffness of composite laminates is rarely considered in current analytical models of curing deformation. Furthermore, the evolution of the interaction between the tool and the composite parts with the degree of cure of the resin has rarely been considered.

Inspired by the abovementioned facts, in this paper, we propose an analytical model to study the curing deformation of composite materials considering coupling stiffness. Based on the CLT, the thermal deformation of composites during the curing process is calculated by considering the evolution of the mechanical properties of resins. A modified adhesive shear layer theory is utilized to simulate the variation of the tool–part interaction with resin curing. Finally, the effects of the curing cycles, geometry, and tool thermal expansion on the curing deformation of the composite are investigated and discussed.

## 2. Theoretical Formulations

The cure reaction of resin can be characterized by a cure kinetics model, which is a function of temperature and the degree of cure. The mathematical cure kinetics model is expressed as:

(1) $$\frac{d\alpha }{dt}=A_{0}\exp\left(\frac{-E_{a}}{RT}\right)\alpha^{m}{\left(1-\alpha \right)}^{n}$$

where α is the degree of cure, *A*_0_ is the pre-exponential constant, *E_a_* is the activation energy, *R* is the universal gas constant, *T* is the temperature, and *m* and *n* are the orders of reaction.

During the curing process, the resin undergoes a change of state from viscous flow, rubbery to a glassy state, which leads to a significant increase in mechanical properties. The resin modulus develops over time and can be modeled in a cure-hardening instantaneously linear elastic model (CHILE), as in [42].

(2) $$E_{m}=(1-\alpha_{\mathrm{mod}})E_{m}^{0}+\alpha_{\mathrm{mod}}E_{m}^{\infty }$$

(3) $$\alpha_{\mathrm{mod}}=\frac{\alpha -\alpha_{gel}}{1-\alpha_{gel}}$$

where $E_{m}^{0}$ and $E_{m}^{\infty }$ are the resin moduli of uncured and fully cured resin, respectively; and $\alpha_{gel}$ is the gel point degree of the cure. By using the self-consistent micromechanics model [43], the effective mechanical properties of the laminate at moment *t* in the curing process are given in Appendix A.

The stress–strain relationship for the *k*th layer of the laminate along any x- and y-axis during the curing process can be expressed as:

(4) $$\left\{\sigma \right\}=\left[{\bar{Q}}_{ij}\right]\left\{\varepsilon^{Total}-\varepsilon_{}^{Th}-\varepsilon_{}^{Sh}\right\}$$

where $\varepsilon^{Total}$, $\varepsilon_{}^{Th}$, and $\varepsilon_{}^{Sh}$ refer to the mechanical strain, thermal expansion strain, and cure shrinkage strain, respectively; and ${\bar{Q}}_{ij}$ is the lamina modulus matrix at the current temperature and time, as listed in Appendix A.

The thermal expansion strain in each direction can be expressed as:

(5) $$\varepsilon_{i}^{Th}=\beta_{i}\Delta T$$

where β is the coefficient of thermal expansion (CTE) of the composite laminate; and ΔT represents the temperature increment and can be obtained from $\Delta T=T_{t}-T_{t+\Delta t}$, in which *t* and Δt denote curing time and a tiny increment in time, respectively.

It is assumed that the chemical shrinkage of resin is the same in all directions during the curing process. Then, the curing shrinkage strain of the resin can be obtained as:

(6) $$\begin{array}{l} \varepsilon_{m}^{Sh}=\sqrt[{3}]{1+\Delta V_{{}^{m}}^{Sh}}-1\\ \Delta V_{m}^{Sh}=\Delta \alpha V_{Sh,\;m}^{total} \end{array}$$

where $\Delta V_{{}^{m}}^{Sh}$ is the volume shrinkage of the resin, $V_{Sh,\;m}^{total}$ is the total volume shrinkage of the resin when fully cured, and Δα is the curing degree increment.

To obtain the stresses resulting from the cure shrinkage of resin, the effective shrinkage strains of the lamina must first be determined. It is assumed that the volume of the fiber does not shrink during curing. According to the mixing law, the effective shrinkage strains of composite material can be expressed as [44]:

(7) $$\begin{array}{l} \varepsilon_{1}^{Sh}=\frac{\varepsilon_{m}^{Sh}E_{m}\left(1-V_{f}\right)}{E_{1f}V_{f}+E_{m}\left(1-V_{f}\right)}\\ \varepsilon_{2}^{Sh}=\varepsilon_{3}^{Sh}=\left(\varepsilon_{m}^{Sh}+\upsilon_{m}\varepsilon_{m}^{Sh}\right)\left(1-V_{f}\right)-[\upsilon_{12f}V_{f}+\upsilon_{m}\left(1-V_{f}\right)]\frac{\varepsilon_{m}^{Sh}E_{m}\left(1-V_{f}\right)}{E_{1f}V_{f}+E_{m}\left(1-V_{f}\right)} \end{array}$$

where the subscripts *f* and *m* denote fiber and resin, respectively; υ is the Poisson’s ratio; and $V_{f}$ is the fiber volume fraction of the composite.

### 2.1. Curing Deformation without Consideration of Tool Constraints

**Figure 1:**
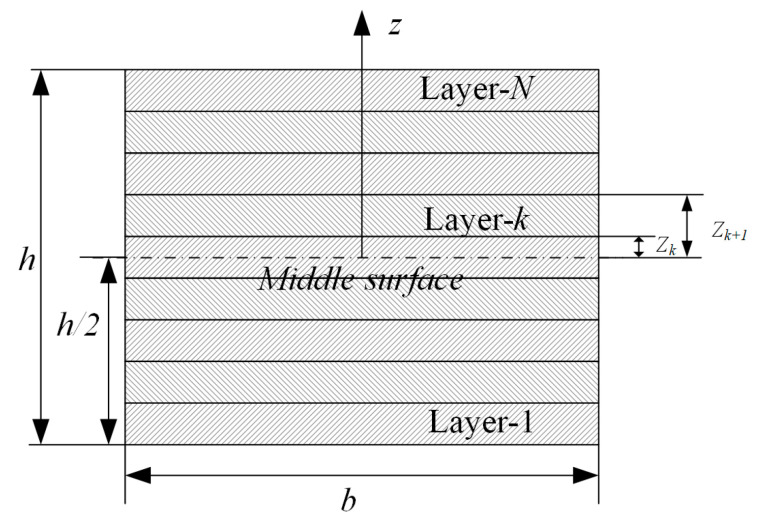
Lay-up of the laminated composite.

Considering that conventional laminates are thin, it is assumed that the temperature distribution in the laminate is uniform. As illustrated in Figure 1, according to the CLT, the incremental internal forces (*N*) and moments (*M*) per unit width in the laminate resulting from the curing process can be expressed as

(8) $$\begin{array}{l} \Delta N=\int \sigma_{k}dz=\sum_{k=1}^{N}\left[\int_{z_{k-1}}^{z_{k}}\sigma_{k}dz\right]\\ \Delta M=\int \sigma_{k}zdz=\sum_{k=1}^{N}\left[\int_{z_{k-1}}^{z_{k}}\sigma_{k}zdz\right] \end{array}$$

Considering Equations (4) and (5) and $\varepsilon_{k}^{Total}=\varepsilon^{0}+z\kappa$, Equation (8) at time *t* of the curing process can be rewritten as:

(9) $$\begin{array}{l} \Delta N=\sum_{k=1}^{N}\left[\int_{z_{k-1}}^{z_{k}}{\bar{Q}}_{k}\left(\varepsilon^{0}+z\kappa -\beta_{k}\Delta T-\varepsilon_{k}^{Sh}\right)\right]dz\\ \Delta M=\sum_{k=1}^{N}\left[\int_{z_{k-1}}^{z_{k}}{\bar{Q}}_{k}\left(\varepsilon^{0}+z\kappa -\beta_{k}\Delta T-\varepsilon_{k}^{Sh}\right)\right]zdz \end{array}$$

where $\varepsilon^{0}$ and κ denote the mid-plane strain and curvature, respectively.

The composite laminate is restrained to the tool during curing, and the surface is covered with vacuum bags. Therefore, it can be assumed that $\varepsilon^{0}$ = κ = 0 in Equation (9). The incremental internal forces ($\Delta N^{E}$) and moments ($\Delta M^{E}$) due to the temperature and cure shrinkage of resin can be recalculated as:

(10) $$\begin{array}{l} \Delta N^{E}=\int {\bar{Q}}_{k}\left(\beta_{k}\Delta T+\varepsilon_{k}^{Sh}\right)dz\\ \Delta M^{E}=\int {\bar{Q}}_{k}\left(\beta_{k}\Delta T+\varepsilon_{k}^{Sh}\right)zdz \end{array}$$

### 2.2. Curing Deformation Considering Mold Constraints

According to Arafath’s work [37], the interaction between the tool and the laminate part can be simulated by an adhesive shear layer, which is located on the bottom of the part close to the tool. The adhesive shear layer is very thin and can only transmit shear stresses along its thickness. In this model, the interfacial shear stress is described as a function of the degree of cure and reflects the change in tool–part interaction during the curing process. The interfacial shear stress is given by:

(11) $$\tau_{s}=G_{s}^{c}\frac{u_{t}-u_{p}^{1}}{h_{s}}$$

(12) $$G_{s}^{c}=(1-\alpha_{\mathrm{mod}})G_{s}^{0}+\alpha_{\mathrm{mod}}G_{s}$$

where $G_{s}^{c}$ denotes the shear modulus of the adhesive layer during curing; $G_{s}^{0}$ and $G_{s}$ represent shear moduli of the adhesive layer in the uncured and fully cured state, respectively; $h_{s}$ is the thickness of the adhesive layer; $\alpha_{\mathrm{mod}}$ is defined in Equation (3); and $u_{t}$ and $u_{p}^{1}$ are the longitudinal displacements of the tool and the layer close to the tool, respectively.

**Figure 2:**
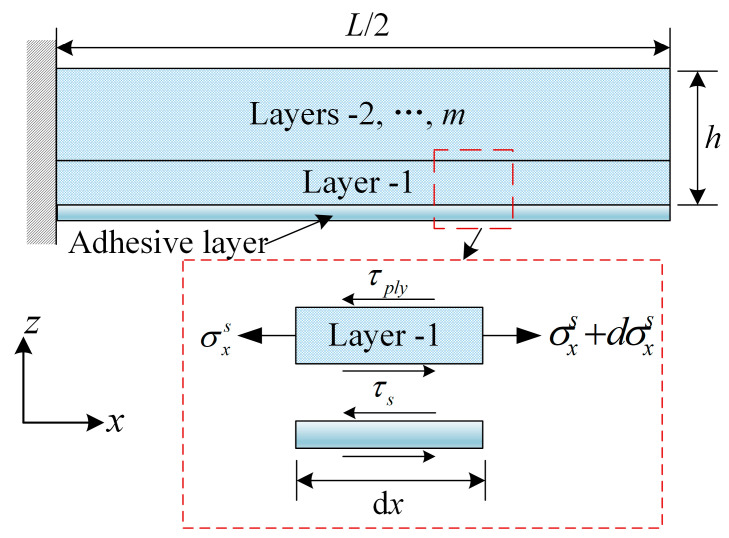
Schematic of laminated beam under shear traction due to tool expansion.

Assuming that there is no stress gradient along the thickness resulting from tool–part interaction and residual stresses caused by tool thermal expansion are only generated in the first layer, as shown in Figure 2, the equilibrium equation of the unit width force for the first layer is expressed as:

(13) $$\left(\sigma_{x}^{S}+d\sigma_{x}^{S}-\sigma_{x}^{S}\right)h_{ply}+\left(\tau_{s}-\tau_{ply}\right)dx=0$$

(14) $$d\sigma_{x}^{S}=\frac{\tau_{ply}-\tau_{s}}{h_{ply}}dx\;$$

where $h_{ply}$ is the is the thickness of the first layer of the composite. Considering $\sigma_{x=L}^{S}=0$, the in-plane stress in the first ply of laminated part is:

(15) ∫0σdσxS=∫L2xτply−τshplydx

(16) $$\sigma_{x}^{S}=\frac{\tau_{ply}-\tau_{s}}{h_{ply}}\left(\frac{L}{2}-x\right)$$

It is assumed that $\tau_{ply}$ = 0 under the effect of tool expansion only, without taking into account the temperature-induced thermal stresses inside the lamina during the curing process. Substituting Equation (11) into Equation (16):

(17) $$\sigma_{x}^{S}=-G_{s}^{c}\frac{u_{t}-u_{p}^{1}}{h_{s}t_{ply}}\left(\frac{L}{2}-x\right)$$

By the same token, the stress ($\sigma_{y}^{S}$) in the y-direction caused by the tool thermal expansion can be obtained as:

(18) $$\sigma_{y}^{S}=-G_{s}^{c}\frac{u_{t,y}-u_{p,y}^{1}}{h_{s}t_{ply}}\left(\frac{L_{y}}{2}-y\right)$$

where the subscript *y* indicates the value of the parameters *u*, *L*, and *σ* in the *y* direction. Therefore, the incremental effective internal forces and moments for the composite laminate considering the interaction between tool and part are:

(19) $$\begin{array}{l} \Delta N^{E}=\int \left[{\bar{Q}}_{k}\left(\beta_{k}\Delta T+\varepsilon_{k}^{Sh}\right)+\sigma^{S}\right]dz\\ \Delta M^{E}=\int \left[{\bar{Q}}_{k}\left(\beta_{k}\Delta T+\varepsilon_{k}^{Sh}\right)+\sigma^{S}\right]zdz \end{array}$$

Small time steps are used to calculate the incremental and instantaneous moment. The total effective forces and moments at curing time *t* are:

(20) $$\begin{array}{l} {N^{E}|}_{t+\Delta t}={N^{E}|}_{t}+\Delta N^{E}\\ {M^{E}|}_{t+\Delta t}={M^{E}|}_{t}+\Delta M^{E} \end{array}$$

After the composite part has been released from the tool, it can obtain the in-plane strain and curvature as:

(21) $$\left\{\begin{array}{c} \Delta \varepsilon \\ \Delta \kappa \end{array}\right\}={\left[\begin{array}{cc} A^{\prime } & B^{\prime }\\ B^{\prime } & D^{\prime } \end{array}\right]}^{End}\left\{\begin{array}{c} N^{End}\\ M^{End} \end{array}\right\}$$

where ***A***, ***B***, and ***D*** are the extensional, coupling and bending stiffness matrices and can be calculated with the elastic moduli at the end of the curing cycle as:

(22) $${\left(\begin{array}{ccc} A_{ij} & B_{ij} & D_{ij} \end{array}\right)}^{End}=\int_{-h/2}^{h/2}{\bar{Q}}_{ij}^{k}\left(\begin{array}{ccc} 1 & z & z^{2} \end{array}\right)dz=\sum_{k=1}^{N}\int_{z_{k-1}}^{z_{k}}{\bar{Q}}_{ij}^{k}\left(\begin{array}{ccc} 1 & z & z^{2} \end{array}\right)dz$$

Once removed from the tool, the part is free to deform. Therefore, a cantilever beam model of 1/2 the length of the composite part is assumed. According to the strain and curvature in Equation (21), the curing deformation of the composite part can be obtained.

## 3. Results and Discussion

In this section, the accuracy of the proposed analytical model is first verified. Then, a comprehensive parametric study is carried out to investigate the influences of curing cycles, geometry, and tool thermal expansion on the curing deformation of the composite part. The finite element software ABAQUS can be used to calculate the curing deformation of composite parts and compare it with the analytical method proposed in this paper. The curing deformation calculation in FEM includes thermochemical and thermomechanical analyses; further details can be found in [45,46].

### 3.1. Comparison Studies

The material properties for the composite part made from AS4/8552 are listed in Table 1. The cure kinetics parameters employed in Equation (1) are as follows [47]: *A*_0_ = 4.2 × 10^6^/min, *E_a_* = 6.5 × 10^4^ J/mol, *m* = 0.5, and *n* = 1.5. The recommended curing cycle consists of a ramp of 2 °C/min heating to the first dwelling temperature of 120 °C from room temperature (20 °C) and holding for 60 min. Then, the temperature is increased to the cure temperature of 180 °C at a heating rate of 2 °C/min and held for 120 min. Finally, the composite is cold to room temperature at a ramp of 2 °C/min.

A comparison of the maximum curing deformation obtained by the present analytical method with the results from FEM is given in Table 2. The geometric dimensions of the composite part used in this case are: length (*L*) = 200 mm and width (*b*) = 25 mm. The shear modulus and thickness of the adhesive layer are: *G*_s_ = 12 kPa, *h_s_* = 0.15 mm. The curing deformations determined by the analytical model are in agreement with the FEM results.

**Figure 3:**
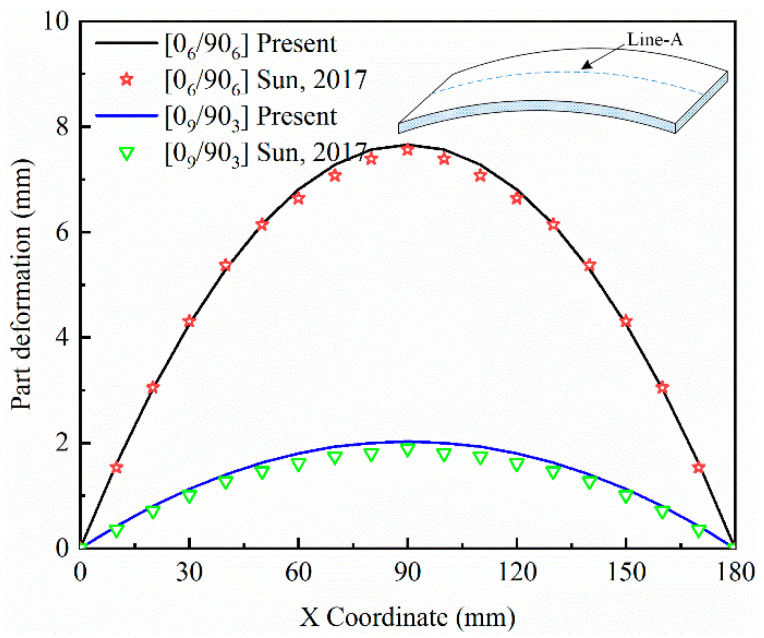
Comparison of the deformation profiles for the present model with those by Sun et al. Reprinted with permission from [36]. Copright 2017 Elsevier Ltd.

Figure 3 shows the deformation fields of two types of laminated composite parts along the *x* direction. The deformation values at the position Line-A are adopted. The deformation fields obtained by the present method are compared with those obtained by Sun et al. [36]. In this case, the dimensions are: length (*L*) = 180 mm and width (*b*) = 20 mm for the [0_6_/90_6_] and [0_9_/90_3_] composite parts. The present results and those reported in Ref. [36] are in agreement.

Based on this verification of the reliability of the analytical model, we conclude that it is suitable for investigating the curing deformation of laminate composite parts. Therefore, the influences of different factors, such as curing cycles, geometry, resin properties, and tool thermal properties, on curing deformation will be investigated using the analytical model.

### 3.2. Parametric Analysis

#### 3.2.1. Influence of Geometric Dimensions

**Figure 4:**
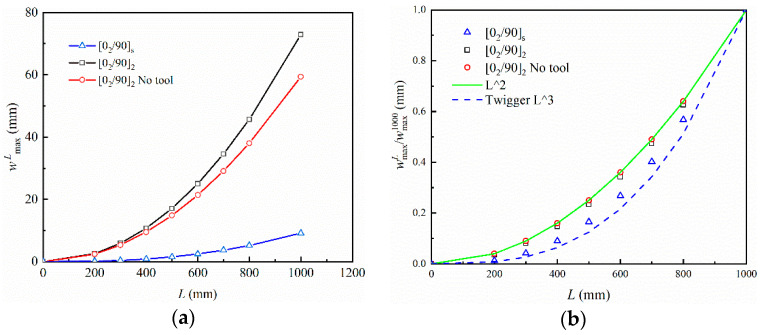
Influence of part length on the maximum curing deformation: (**a**) maximum deformation and (**b**) normalized maximum deformation.

Figure 4 depicts the relationship between part length and curing deformation of composite parts compared with the pattern predicted by Twigg [38,48]. $w_{\max}^{L}$ normalized to the $w_{\max}^{1000}$ under the same curing conditions, where $w_{\max}^{L}$ refers to the maximum curing deformation of parts with different lengths. Laminated parts with both symmetrical and asymmetrical lay-ups are considered, as well as when the tool–part interaction is not taken into account. The length of the part has a significant influence on the maximum curing deformation, which increases as a power function of the part length. Twigg pointed out that the maximum curing deformation of a part is proportional to the third power of the length. In the case of parts with symmetrical lay-up, the relationship between the maximum curing deformation and the length of the part satisfies the law, as shown in Figure 4b. However, the results are not similar for parts with asymmetrical lay-up. If the contribution of tool–part interaction to curing deformation is ignored, the maximum deformation is proportional to the square of the part length.

**Figure 5:**
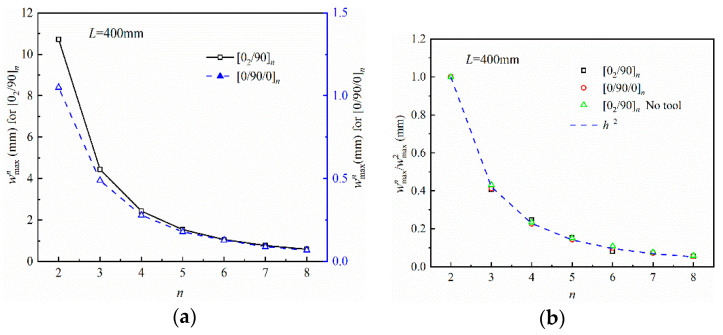
Influence of part thickness on the maximum curing deformation: (**a**) maximum deformation and (**b**) normalized maximum deformation.

Figure 5 shows the influence of thickness on the maximum curing deformation of the composite part. Parts with [0_2_/90]*_n_* and [0/90/0]*_n_* lay-up types are considered, where *n* = 2, …, 8 in this case. $w_{\max}^{n}$ normalized to the $w_{\max}^{2}$ under the same curing conditions, where $w_{\max}^{n}$ refers to the maximum curing deformation of parts with different layers. The curing deformation decreases as the thickness increases, and the maximum curing deformation versus thickness follows the trend of 1/*h*^2^, independent of the lay-up sequence and tool–part interaction.

#### 3.2.2. Influence of Curing Temperature Cycles

**Figure 6:**
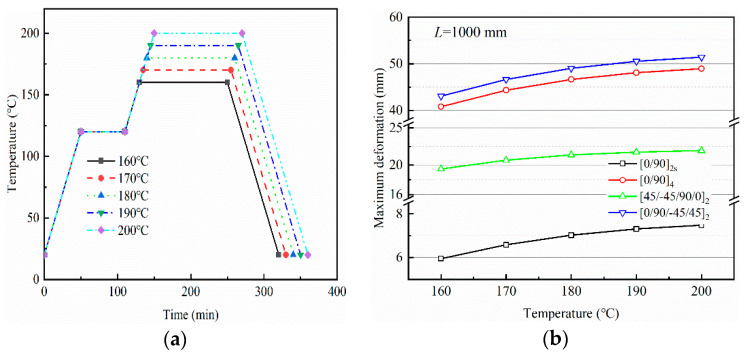
Influence of curing temperature on the maximum deformation of parts with different lay-up sequences: (**a**) curing cycles and (**b**) maximum deformation.

**Figure 7:**
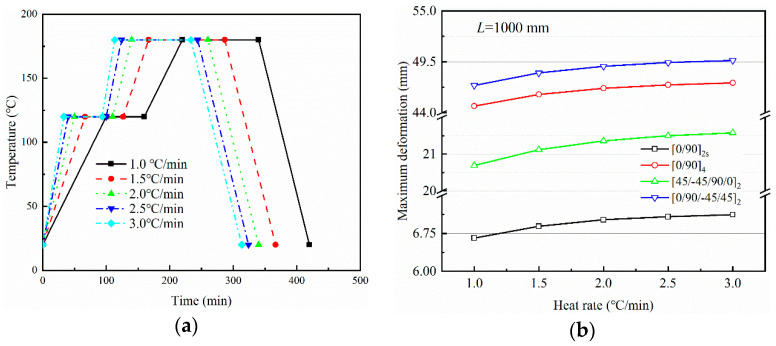
Influence of heating rate on the maximum deformation of parts with different lay-ups: (**a**) curing cycles and (**b**) maximum deformation.

Figure 6 and Figure 7 show the variation of maximum curing deformation of a composite part for *L* = 1000 mm with curing temperature and heating rate, respectively. A curing temperature of *p* °C (*p* = 160, …, 180) and heating rate of *q* °C/min (*q* = 1.0, …, 3.0) are taken into consideration, and the curing cycles are shown in Figure 6a and Figure 7a. Curing deformation increases with increased curing temperature or heating rate, and the pace of growth slows. For fully elastic composite materials, the part deformation returns to its original state after heating and cooling. However, the tensile action of the tool on the part and the inconsistent thermal deformation of each layer result in strain gradients that gradually freeze together as the resin cures. When curing is completed, warpage deformation occurs. The non-linear variation of curing deformation with curing temperature is due to an increase in temperature and heating rate, which accelerates the curing reaction and brings the resin into its elastic state earlier. The resin curing reaction slows down at a later stage, when the resin is nearing the end of its cure.

#### 3.2.3. Influence of Resin Characteristics

**Figure 8:**
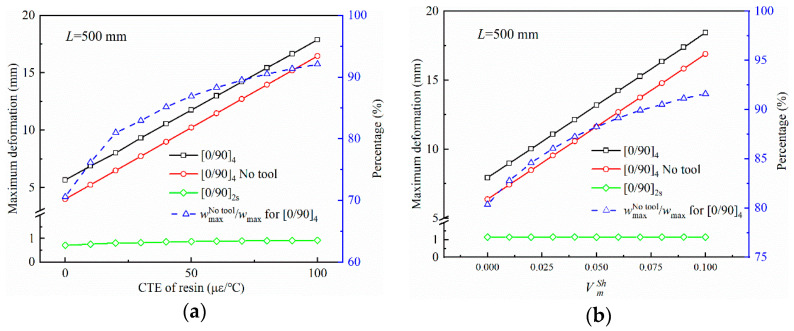
Influence of resin characteristics on the maximum deformation of parts: (**a**) thermal expansion coefficient of resin and (**b**) curing shrinkage of the resin volume.

Figure 8 illustrates the influence of resin characteristics, including thermal expansion and curing shrinkage, on the curing deformation of composite parts. The material parameters are the same as in Section 3.1 except for the thermal expansion and curing shrinkage. The curing deformation of parts with asymmetric lay-ups increases linearly with the coefficient of thermal expansion and volume shrinkage of the resin. This can be attributed to the thermal expansion and curing volume shrinkage of the resin making the anisotropy of the laminate more pronounced and the variation in deformation of each layer with increased temperature. However, for symmetrically layered parts, the curing deformation remains constant, entirely due to the traction of the tool on the part during the curing process. The proportion of curing deformation to total curing deformation without taking into account the action of the tool increases gradually with the increase in the thermal expansion coefficient and volume shrinkage of the resin, with a slowed pace of increase.

#### 3.2.4. Influence of Tool Thermal Expansion

**Figure 9:**
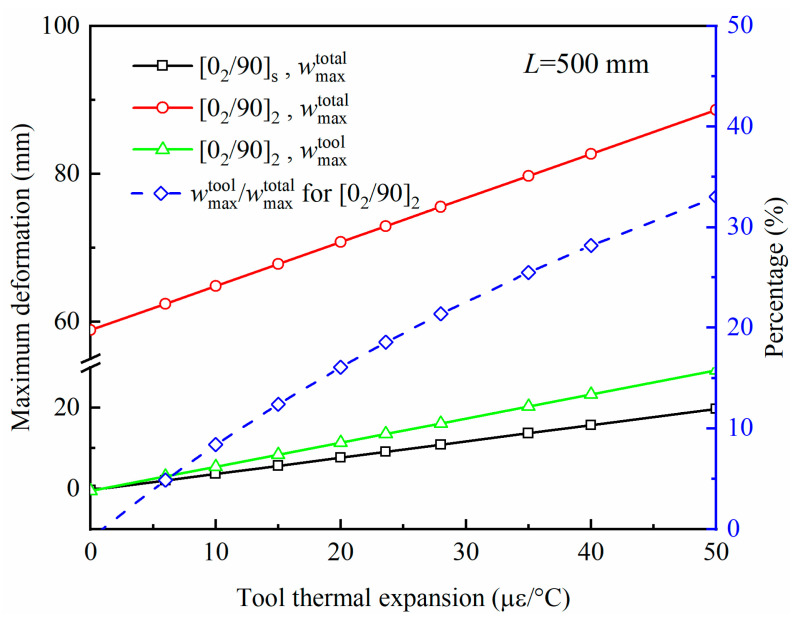
Variation of curing deformation with the coefficient of thermal expansion of the tool.

The relationship between the tool thermal expansion and the deformation of the composite part with *L* = 500 mm is shown in Figure 9. The material parameters are the same as in Section 3.1, except the thermal expansion coefficient of the. $w_{\max}^{\mathrm{tool}}$ and $w_{\max}^{\mathrm{total}}$ indicate the curing deformation when only the tool–part interaction is applied and the actual total curing deformation, respectively. The curing deformation of the composite part increases linearly with the thermal expansion coefficient of the tool. For [0_2_/90]_2_ laminated parts, the percentage of the tool–part interaction in the actual curing deformation increases with the thermal expansion coefficient of the tool, but the increase becomes progressively slower. This is because the curing deformation caused by other factors, such as stack sequence, thermal expansion, etc., remains constant.

**Figure 10:**
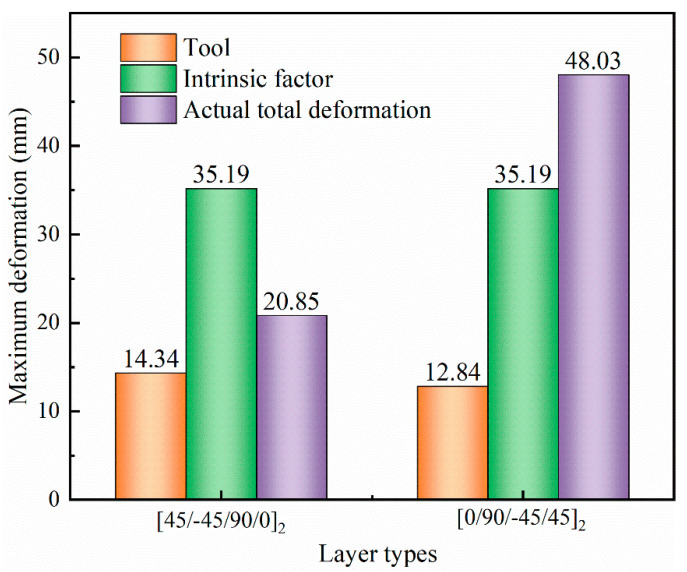
Influence of tool expansion on the curing deformation of parts.

**Figure 11:**
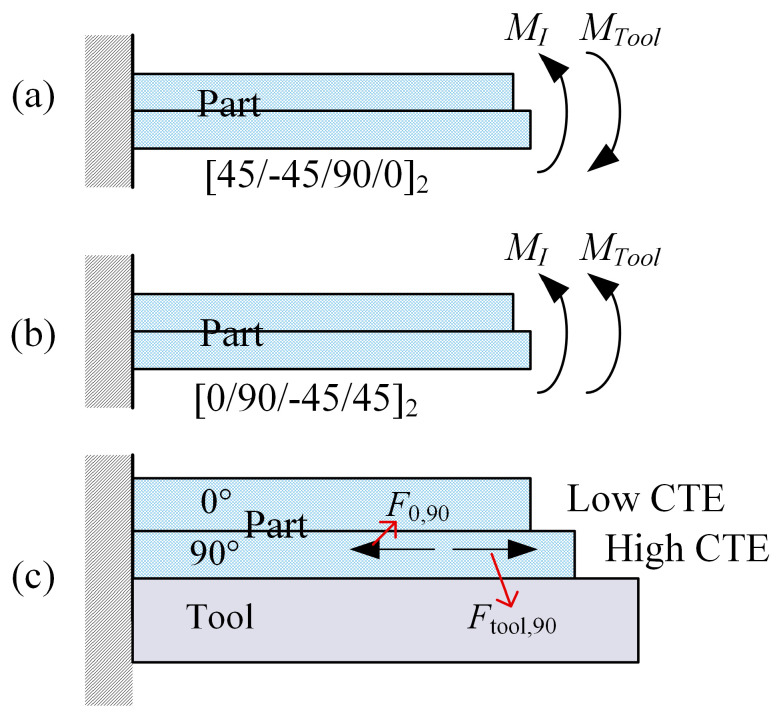
Schematic diagram of the effect of tool and lay-up on curing deformation: (**a**) internal moment of [45/−45/90/0]_2_ laminate part generated from the tool and internal factors; (**b**) internal moment of [0/90/−45/45]_2_ laminate part generated from the tool and internal factors; and (**c**) effect of tool and lay-up on internal forces in asymmetric lay-ups.

The role of the tool in the curing deformation of composite parts with *L* = 1000 mm is further illustrated in Figure 10. Factors influencing curing deformation are divided into intrinsic factors and tool action. Curing deformation caused by intrinsic factors occurs when the curing temperature of the asymmetric composite is lowered to room temperature. The actual curing deformation of [45/−5/90/0]_2_ laminates is much less than that of [0/90/45/−45]_2_ laminates. Without taking into consideration the action of the tool, the curing deformation of the parts is the same for the two lay-up methods, [45/−45/90/0]_2_ and [0/90/45/−45]_2_, with an opposite trend for warpage. This means that the curing deformation differs considerably for asymmetric composite parts with opposite lay-up sequences. This is determined by the direction of the internal moment generated by the stretching of the part by the composite lay-up and the mold during curing. A schematic illustration is presented in Figure 11. *M_I_* and *M_Tool_* represent the internal moments generated by internal factors and tool stretching in composite parts, respectively. *F*_0,90_ and *F*_tool,90_ denote the acting force of a 0° layer and a tool on a 90° layer, respectively. Given $\beta_{tool}$ > $\beta_{layer}^{90}$ > $\beta_{layer}^{0}$, the tool and the 0° layer have opposite directions of force on the 90° layer. For the same reason, the force of the top layers of [0/90/−45/45]_2_ laminate on the bottom layers is in the same direction as the force of the tool on the bottom layers. However, the opposite is true for [45/−45/90/0]_2_. This indicates that the thermal expansion of the tool has a suppressive effect on the curing deformation of [45/−45/90/0]_2_ laminate, while enhancing the curing deformation of [0/90/−45/45]_2_ laminate.

#### 3.2.5. Contribution of Different Factors to Curing Deformation

**Figure 12:**
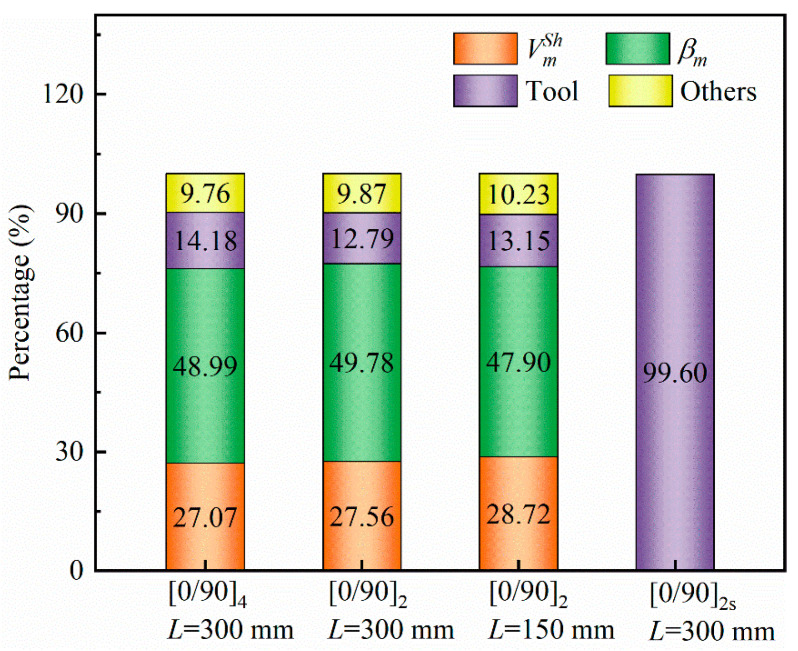
Contribution of different factors to curing deformation of the parts.

Figure 12 shows the contribution of curing shrinkage, thermal expansion of the resin, tool–part interaction, and other factors to the curing deformation of the composite during a given curing cycle. Material parameters are the same as in Section 3.1. The percentage of different factors is equal to $1-w_{\max}^{P=0}/w_{\max}^{\mathrm{total}}$, where P = 0 (P stands for $V_{m}^{Sh}$, $\beta_{m}$ and Tool) means that the value of the selected factor is 0 (e.g., $V_{m}^{Sh}$ = 0). The interaction between the tool and the part plays an almost total role in the curing deformation of symmetrical parts. For asymmetrical parts, the thermal expansion of the resin plays the most important role, followed by resin curing shrinkage, tool–part interaction, and other factors. For non-symmetrical parts, the weight of different factors in curing deformation has little correlation with geometry and lay-up. In order to reduce the curing deformation of the composite, symmetrically layered parts are the better choice. For asymmetrically layered parts, it is also of considerable interest to reduce the thermal expansion coefficient and curing volume shrinkage of the resin.

## 4. Conclusions

Based on classical laminate heat deformation theory, in this paper, we present an analytical model that takes into account resin material evolution and part–tool interactions to investigate the curing behavior of composite parts during the curing process. An interface layer is used to simulate the traction effect of the tool on the parts during the curing process, the shear modulus of which is defined as a function of the degree of cure of the resin. A comprehensive parametric study was conducted, and the results show that:

1. The analytical model proposed in this work can deal with the issues of process-induced stresses and deformations during the curing process of composite parts, considering the coupled stiffness of laminate composites. The results of the model can be trusted in comparison with the results of previous literature and FEM. The analytical model makes it easier and faster to analyze curing behavior and provides a deeper understanding of the physical mechanisms of curing deformation in composites. The influence of different factors on curing deformation can be evaluated individually.
2. Geometry has a considerable influence on the curing deformation of composite parts. However, for a composite part with definite geometric dimensions, curing deformation is largely attributed to the tool–part interaction, curing shrinkage, and high thermal expansion of the resin. For composite parts with asymmetrical lay-up, the curing deformations of composite parts are mainly due to their own anisotropy, including the anisotropy of each layer of laminate in macroscopic aspect, and the anisotropy of resin and fiber in microscopic aspect. The enhancement and suppression of curing deformation by the tool–part interaction depend on the lay-up sequence. The curing deformation of symmetrically layered parts is mainly due to the influence of the tool.
3. From the point of view of reducing curing distortion, composites with low curing shrinkage and CTE resins are the best choices, followed by a tool with low-thermal-expansion material. Adjustments to the cure cycle can improve manufacturing accuracy to a certain extent. Finally, tool compensation may be required to achieve zero curing deformation.

## Figures and Tables

**Table 1:** Material properties of AS4/8552 [36]. Reprinted with permission from Ref. [36]. Copyright 2017 Elsevier Ltd.

Properties	AS4 Fiber	8552
*E*_1_ (GPa)	207.0	4.67
*E*_2_ = *E*_3_ (GPa)	20.4	-
*G*_12_ = *G*_13_ (GPa)	27.6	1.7
*G*_23_ (GPa)	6.89	-
$\upsilon_{12}$ = $\upsilon_{13}$	0.2	0.37
$\upsilon_{23}$	0.3	-
$\beta_{1}$ (με/°C)	−0.9	48.7
$\beta_{2}$ = $\beta_{3}$ (με/°C)	7.2	-

**Table 2:** Comparison of maximum curing deformation.

Lay-Up	Maximum Curing Deformation		
	Present (mm)	FEM (mm)	Difference
[0_6_/90_6_]	9.31	8.81	5.37%
[0_9_/90_3_]	2.47	2.66	7.69%
[0_3_/90]_2_	1.30	1.32	1.54%
[0_3_/90]_s_	0.17	0.16	5.88%
[0/90]_s_	0.29	0.30	3.44%

## Data Availability

Data sharing not applicable.

## Appendix A

The lamina modulus matrix at a given time during the curing process can be expressed as:

(A1) $$\begin{array}{l} {\bar{Q}}_{11}^{k}=Q_{11}^{k}c^{4}+2\left(Q_{12}^{k}+2Q_{66}^{k}\right)c^{2}s^{2}+Q_{22}^{k}s^{4}\\ {\bar{Q}}_{12}^{k}=\left(Q_{11}^{k}+Q_{22}^{k}-4Q_{66}^{k}\right)c^{2}s^{2}+Q_{12}^{k}\left(c^{4}+s^{4}\right)\\ {\bar{Q}}_{22}^{k}=Q_{11}^{k}s^{4}+2\left(Q_{12}^{k}+2Q_{66}^{k}\right)c^{2}s^{2}+Q_{22}^{k}c^{4}\\ {\bar{Q}}_{16}^{k}=\left(Q_{11}^{k}-Q_{12}^{k}-2Q_{66}^{k}\right)c^{3}s+\left(Q_{12}^{k}-Q_{22}^{k}+2Q_{66}^{k}\right)cs^{3}\\ {\bar{Q}}_{26}^{k}=\left(Q_{11}^{k}-Q_{12}^{k}-2Q_{66}^{k}\right)cs^{3}+\left(Q_{12}^{k}-Q_{22}^{k}+2Q_{66}^{k}\right)c^{3}s\\ {\bar{Q}}_{44}^{k}=Q_{44}^{k}c^{2}+Q_{55}^{k}s^{2}\\ {\bar{Q}}_{45}^{k}=\left(Q_{55}^{k}-Q_{44}^{k}\right)cs\\ {\bar{Q}}_{55}^{k}=Q_{55}^{k}c^{2}+Q_{44}^{k}s^{2} \end{array}$$

where $c=\cos\theta_{f}^{k}$ and $s=\sin\theta_{f}^{k}$. The lamina elastic coefficients $Q_{ij}^{k}$ at a moment during curing process are obtained from the material properties of the *k*th layer:

(A2) $$\begin{array}{l} Q_{11}^{k}=\frac{E_{11}^{k}}{1-\upsilon_{12}^{k}\upsilon_{21}^{k}},Q_{12}^{k}=\frac{\upsilon_{12}^{k}E_{22}^{k}}{1-\upsilon_{12}^{k}\upsilon_{21}^{k}},Q_{22}^{k}=\frac{E_{22}^{k}}{1-\upsilon_{12}^{k}\upsilon_{21}^{k}}\\ \;\;\;\;\;\;\;\;\;Q_{44}^{k}=G_{23}^{k},Q_{55}^{k}=G_{13}^{k},Q_{66}^{k}=G_{12}^{k} \end{array}$$

where $E_{1}^{k}$, $E_{2}^{k}$, $G_{12}^{k}$, $G_{13}^{k}$, and $G_{23}^{k}$ are the tensile elastic moduli and the shear moduli *k*th layer of the composite at a certain point in the cure cycle. The effective mechanical properties of the composite properties can be obtained by [43].

(A3) $$E_{11}=E_{1f}V_{f}+E_{m}\left(1-V_{f}\right)+\left[\frac{4\left(\upsilon_{m}-\upsilon_{12f}^{2}\right)K_{f}K_{m}G_{m}\left(1-V_{f}\right)V_{f}}{\left(K_{f}+G_{m}\right)K_{m}+\left(K_{f}-K_{m}\right)G_{m}V_{f}}\right]$$

(A4) $$E_{22}=E_{33}=\frac{1}{\left(1/4K_{T}\right)+\left(1/4G_{23}\right)+\left(\upsilon_{12}^{2}/E_{11}\right)}$$

(A5) $$G_{12}=G_{13}=G_{m}\left[\frac{\left(G_{12f}+G_{1m}\right)+\left(G_{12f}-G_{m}\right)V_{f}}{\left(G_{12f}+G_{m}\right)-\left(G_{12f}-G_{m}\right)V_{f}}\right]$$

(A6) $$G_{23}=\frac{G_{m}\left[K_{m}\left(G_{m}+G_{23f}\right)+2G_{m}G_{23f}+K_{m}\left(G_{23f}-G_{m}\right)V_{f}\right]}{K_{m}\left(G_{m}+G_{23f}\right)+2G_{m}G_{23f}-\left(K_{m}+2G_{m}\right)\left(G_{23f}-G_{m}\right)V_{f}}$$

(A7) $$\upsilon_{12}=\upsilon_{13}=\upsilon_{12f}V_{f}+\upsilon_{m}\left(1-V_{f}\right)+\left[\frac{\left(\upsilon_{m}-\upsilon_{12f}\right)\left(K_{m}-K_{f}\right)G_{m}\left(1-V_{f}\right)V_{f}}{\left(K_{f}+G_{m}\right)K_{m}+\left(K_{f}-K_{m}\right)G_{m}V_{f}}\right]$$

(A8) $$\upsilon_{23}=\frac{2E_{11}K_{T}-E_{11}E_{22}-4\upsilon_{12}^{2}K_{T}E_{22}}{2E_{11}K_{T}}$$

(A9) $$K_{f}=\frac{E_{11f}}{2\left(1-\upsilon_{12f}-2\upsilon_{12f}^{2}\right)}$$

(A10) $$K_{m}=\frac{E_{m}}{2\left(1-\upsilon_{m}-2\upsilon_{m}^{2}\right)}$$

(A11) $$K_{T}=\frac{\left(K_{f}+G_{m}\right)K_{m}+\left(K_{f}-K_{m}\right)G_{m}V_{f}}{\left(K_{f}+G_{m}\right)-\left(K_{f}-K_{m}\right)V_{f}}$$

(A12) $$\beta_{11}=\frac{\beta_{fl}E_{f}V_{f}+\beta_{m}E_{m}V_{m}}{E_{f}V_{f}+E_{m}V_{m}}$$

(A13) $$\beta_{22}=\beta_{33}=\left(1+\upsilon_{f}\right)\frac{\beta_{fl}+\beta_{fm}}{2}V_{f}+\left(1+\upsilon_{m}\right)\beta_{m}V_{m}-\beta_{11}\upsilon_{12}$$
